# Is Standard TRUS-Guided Prostate Biopsy the End of the Game?

**DOI:** 10.5152/tud.2026.25103

**Published:** 2026-03-13

**Authors:** Erdal Benli, Nurullah Kadim, Ahmet Yüce, Abdullah Çırakoğlu, Mevlüt Keleş, Ayhan Arslan

**Affiliations:** Department of Urology, Ordu University Faculty of Medicine, Ordu, Türkiye

**Keywords:** Cost analysis, MRI-targeted biopsy, prostate cancer, transrectal ultrasound guided biopsy

## Abstract

**Objective:**

Prostate cancer (PCa) remains a major cause of morbidity and mortality among men worldwide, and histopathologic tissue sampling plays a pivotal role in diagnosis. Although transrectal ultrasound–guided prostate needle biopsy (TRUS biopsy) has been the standard diagnostic technique for decades, its effectiveness has increasingly been questioned with the widespread adoption of cognitive and magnetic resonance imaging (MRI)–targeted biopsy techniques. The aim of this study was to present the clinical outcomes and costs of TRUS-guided prostate biopsy performed in the clinic and to compare its costs with those of MRI-targeted biopsy.

**Materials and Methods:**

Data from 221 patients who underwent prostate biopsy were retrospectively analyzed. Patient comorbidities, prostate-specific antigen levels, digital rectal examination findings, histopathologic results, and biopsy-related costs were recorded. Cost data for MRI-targeted biopsy were obtained from 9 private hospitals located in different regions of the country.

**Results:**

Prostate cancer was detected in 40.3% (n = 89) of the 221 patients. Clinically significant PCa was identified in 29.9% (n = 66), whereas malignancy could not be excluded in 1.4% (n = 3). The mean cost of TRUS-guided prostate biopsy performed in the clinic was calculated as US $26.8. In contrast, based on data obtained from 9 hospitals, the reported mean cost of MRI-targeted biopsy was US $1449.

**Conclusion:**

Transrectal ultrasound–guided prostate needle biopsy remains an effective and highly cost-efficient diagnostic method in patients with suspected PCa. This technique may be particularly valuable in clinical settings where access to MRI-targeted biopsy is limited.

**Main Points**

- Diagnostic performance of transrectal ultrasound–guided prostate needle biopsy (TRUS-guided) is comparable to targeted biopsy in selected patient populations.
- Transrectal ultrasound–guided prostate needle biopsy offers substantial advantages in cost, accessibility, and procedural simplicity.
- Given the current limitations in access to targeted biopsy technologies, TRUS-guided biopsy is expected to maintain clinical relevance.

## Introduction

Prostate cancer (PCa) is the second most commonly diagnosed malignancy and the fifth leading cause of cancer-related death among men worldwide. In 2022, approximately 1.4 million new cases of PCa and 396 000 related deaths were estimated globally. The prevalence and mortality of PCa vary substantially across geographic regions due to multiple factors. Prostate cancer remains one of the leading causes of cancer-related mortality in men in several regions, particularly in the Americas, Northern and Western Europe, Australia and New Zealand, and the Caribbean.^1^

Although the exact etiology of PCa remains unclear, genetic predisposition, aging, dietary habits, and environmental factors are believed to contribute to its development. Currently, there are no proven dietary or pharmacologic strategies for the primary prevention of PCa. Therefore, clinical efforts are primarily focused on early detection, which is associated with favorable treatment outcomes.^2^ In contrast, delayed diagnosis is associated with significantly increased morbidity and mortality. Consequently, the cornerstone of PCa management is the detection of disease at an early, organ-confined stage.

Despite the absence of diagnostic tests with 100% sensitivity and specificity, several robust diagnostic tools are routinely used in clinical practice, including prostate-specific antigen (PSA) testing, digital rectal examination (DRE), and, more recently, multiparametric magnetic resonance imaging (mpMRI), which has substantially influenced diagnostic pathways. Definitive diagnosis of PCa requires histopathologic confirmation through prostate tissue sampling. Transrectal ultrasound–guided prostate needle biopsy (TRUS-guided biopsy) has long been considered the gold standard diagnostic method and continues to be widely utilized.^3^ This technique offers several advantages for clinicians, including procedural simplicity, ease of implementation, and a relatively short learning curve. From a patient perspective, it is generally well tolerated and cost-effective. The diagnostic yield of TRUS-guided biopsy varies according to PSA levels.^4^^,^^5^ One study reported false-negative rates of up to 49% among patients with elevated PSA levels.^6^ This variability may be attributed to several factors, including tumor invisibility on ultrasound, operator experience, and patient-related characteristics.

With recent technological advances, targeted biopsy techniques have emerged, enabling sampling of suspicious lesions and improving cancer detection rates. However, these modalities are associated with several limitations, including limited accessibility, higher costs, reliance on advanced imaging, and longer procedural times. As a result, widespread implementation remains challenging in many clinical settings. Until these limitations are adequately addressed and broader accessibility is achieved, conventional TRUS-guided biopsy is likely to remain a mainstay diagnostic approach.

The present study was designed to evaluate the clinical outcomes and costs of TRUS-guided prostate biopsy performed as the standard diagnostic method in the clinic and to compare these findings with targeted biopsy techniques. The aim was to present the institutional experience and to assess the cost-effectiveness of TRUS-guided biopsy in contemporary clinical practice.

## Material and Methods

### Study Design and Patient Population

This retrospective study was conducted between January 2018 and November 2023 at the Department of Urology, Ordu University Faculty of Medicine Hospital. The study protocol was approved by the Ordu University Non-Interventional Scientific Research Ethics Committee (Decision No. 71, June 28, 2024) and was conducted in accordance with the principles of the Declaration of Helsinki. Owing to the retrospective design of the study, the requirement for individual informed consent was waived by the ethics committee.

Patient data were obtained through a retrospective review of institutional medical records and were systematically recorded by an experienced investigator. Patients who underwent prostate biopsy due to elevated PSA levels or suspicious DRE findings were included. A PSA threshold of 4 ng/mL was used, and elevated PSA levels were confirmed by at least 2 separate measurements obtained at different time points.

Patients with unknown histopathologic results, those who underwent biopsy at other institutions, patients with transient PSA elevation, and patients with a history of prior prostate biopsy were excluded. Data regarding patient comorbidities, PSA levels, DRE findings, and biopsy results were collected. Multiparametric magnetic resonance imaging findings were not incorporated into the analysis.

Laboratory parameters were analyzed using serum samples obtained from venous blood collected in the morning after at least 10 hours of fasting. Prior to the procedure, all patients were informed in detail about the biopsy procedure and potential complications, and written informed consent was obtained. For patients receiving anticoagulant therapy, relevant specialties were consulted, and anticoagulation was temporarily discontinued in accordance with clinical recommendations before biopsy.

For antibiotic prophylaxis, oral ciprofloxacin 500 mg was administered in 2 doses: 1 hour before the procedure and 12 hours after the procedure. No bowel preparation, including enema, was performed prior to biopsy. All procedures were performed by an experienced urologist.

Cost data for TRUS-guided biopsy were obtained from the hospital billing department. The cost of targeted biopsy was calculated based on the average prices reported by 9 centers performing targeted prostate biopsy in different regions of the country. In public hospitals, targeted biopsy procedures are not routinely performed, and no reimbursement code exists under the National Health Implementation Communiqué (SUT). A single reimbursement code (No. 621330: ultrasound-guided multiple prostate needle biopsy) is currently used for all prostate biopsy procedures.

### Biopsy Technique

All procedures were performed with the patient in the left lateral decubitus position under sterile conditions. Mucosal anesthesia was achieved using rectal lidocaine gel. Periprostatic nerve block was performed bilaterally with 2 mL of 2% lidocaine. A systematic 12-core biopsy was performed using a 22-gauge needle under transrectal ultrasound guidance (Mindray system).

Biopsy specimens were labeled separately and sent for histopathologic evaluation. All patients were observed for a short period following the procedure and discharged if no complications were noted. Patients were thoroughly instructed regarding potential post-procedural complications, including fever, chills, and bleeding, and were advised to return to the hospital if any adverse symptoms occurred. No routine medications were prescribed at discharge. Follow-up visits were scheduled at 1 week post-procedure and again after availability of the pathology results.

### Statistical Analysis

Statistical analyses were performed using IBM SPSS Statistics for Windows, version 28.0 (IBM Corp., Armonk, NY). The distribution of continuous variables was assessed using the Kolmogorov–Smirnov test. Non-normally distributed data were presented as median (interquartile range (IQR)) and compared using the Mann–Whitney *U*-test. Categorical variables were analyzed using the χ^2^ test.

For proportion estimates, 95% CIs were calculated using the exact binomial method. In all analyses, *P*-value <.05 was considered statistically significant.

## Results

A total of 221 patients underwent prostate biopsy. Baseline demographic characteristics were comparable across the study population. Of the patients, 76 (34.4%) presented for routine surveillance, whereas 145 (65.6%) were referred due to lower urinary tract symptoms. Comorbidities included diabetes mellitus in 36 patients (16.2%), pulmonary disease in 14 (6.3%), hypertension in 85 (38.4%), dyslipidemia in 28 (12.6%), psychiatric disorders in 17 (7.6%), and neurologic disorders in 12 (5.4%) (Table 1).

Prostate cancer was detected in 40.3% of patients (95% CI, 33.7%-47.1%), whereas benign pathology was observed in 52.9% (95% CI, 46.1%–59.7%). Atypical small acinar proliferation was identified in 5.4% of patients (95% CI, 2.8%-9.3%), and malignancy could not be excluded in 1.4% (95% CI, 0.3%-3.9%) (Table 2). The rate of clinically significant PCa was 29.9% (95% CI, 24.0%-36.3%), whereas clinically insignificant PCa accounted for 10.4% (95% CI, 6.7%-15.2%).

The overall median PSA level was 7.49 (IQR, 6.15) ng/mL. Median PSA levels were 7.94 (IQR, 4.80) ng/mL in the benign group and 7.84 (IQR, 36.18) ng/mL in the malignant group. No statistically significant difference in PSA levels was observed between groups (*P* = .08) (Table 3).

According to DRE findings, 134 patients (60.7%) had negative results and 87 (39.3%) had positive findings. In the malignant group, 46 patients (51.7%) had positive DRE findings and 43 (48.3%) had negative findings. In the benign group, 36 patients (30.8%) had positive DRE findings and 81 (69.2%) had negative findings. A statistically significant association was observed between positive DRE findings and PCa detection (*P* = .002) (Table 4).

The mean cost of TRUS-guided prostate biopsy performed in the clinic was US $26.8. In contrast, the mean reported cost of targeted biopsy in the country was US $1449 (Table 5).

## Discussion

Definitive diagnosis of PCa relies on histopathologic evaluation of biopsy specimens obtained from the prostate. Although TRUS-guided biopsy has served as the standard diagnostic technique for nearly 4 decades, several advanced targeted biopsy modalities have recently emerged. The primary objective of this study was to evaluate the diagnostic performance and cost profile of TRUS-guided biopsy in contemporary practice and to examine its position relative to newer technologies. In the cohort, PCa was detected in 40.3% of patients, with a clinically significant PCa (csPCa) detection rate of 29.9%. The mean procedural cost was US $26.8, supporting the feasibility of TRUS-guided biopsy as a cost-efficient diagnostic strategy with acceptable clinical performance.

The first TRUS-guided biopsy was performed in 1937, and the technique was standardized in 1987.^7^ Transition from finger-guided blind sampling to ultrasound-guided anatomic targeting substantially improved procedural accuracy and safety. Since then, TRUS-guided biopsy has remained widely adopted due to its short learning curve, low equipment requirements, minimal infrastructure needs, and feasibility in outpatient settings. In routine clinical practice, residents and early career urologists typically achieve procedural competency after a relatively small number of cases, often within 10 to 20 procedures based on institutional experience. Moreover, the procedure can be readily performed using conventional ultrasound systems commonly available in urology clinics, without the need for complex anesthesia techniques or specialized facilities.^8^

Reported cancer detection rates for TRUS-guided biopsy vary widely across the literature. Kam et al^9^ reviewed histopathologic outcomes in 1049 patients and reported a cancer detection rate of 29.2%. Wei et al^10^ analyzed a large cohort of 12 968 patients and observed a detection rate of 36%. Similar findings were reported by Di Franco et al,^11^ with a detection rate of 34.2%. Higher detection rates have also been described in high-volume centers. Klotz et al^12^ reported PCa in 57.4% of patients, with a csPCa rate of 29.7% and a median PSA level of 6.2 ng/mL. The results are consistent with these data, demonstrating a cancer detection rate of 40.3% and a csPCa rate of 29.9%, closely mirroring outcomes reported by experienced centers. Variability in detection rates across studies likely reflects differences in study design, patient selection, operator expertise, and institutional protocols. When performed by experienced operators, TRUS-guided biopsy can achieve high diagnostic yield. Detection rates may further improve with prebiopsy mpMRI; however, mpMRI interpretation remains highly dependent on radiologist expertise. Similarly, because DRE findings are influenced by examiner-dependent variability, elevated PSA levels were used as the primary objective criterion for biopsy indication in the cohort.

TRUS-guided biopsy continues to represent an effective diagnostic modality in centers without access to advanced targeted biopsy infrastructure. Nevertheless, several limitations must be acknowledged. Tumors located in anterior or apical regions may be under-sampled compared with peripheral zone lesions.^13^ Another limitation is the inability to directly visualize suspicious lesions during sampling, which may contribute to false-negative results and uncertainty regarding index lesion targeting in patients undergoing active surveillance. Mufarrij et al^14^ reported Gleason score upgrading in 46% of patients initially classified as low risk based on preoperative TRUS-guided biopsy findings.

Advances in imaging, particularly the integration of mpMRI, have transformed PCa diagnostics. Multiparametric magnetic resonance imaging enables high-resolution visualization of suspicious lesions and risk stratification using the Prostate Imaging Reporting and Data System (PI-RADS), facilitating targeted sampling and improving csPCa detection rates. In selected patients, negative mpMRI findings (PI-RADS ≤2) may obviate the need for biopsy, reducing patient anxiety and procedure-related morbidity. Importantly, mpMRI enhances discrimination between clinically significant and indolent disease.

Several studies have reported favorable outcomes for MRI-targeted biopsy. Tewes et al^15^ reported a cancer detection rate of 39% using MR-TRUS fusion biopsy. Lavaerts et al^16^ observed PCa in 44.1% of 102 patients with mpMRI-visible lesions, with a csPCa rate of 27.4%. Filson et al^17^ reported a csPCa rate of 31%, whereas Del Monte et al^18^ demonstrated a PCa detection rate of 49% and a csPCa rate of 30.6%, with a median PSA of 7.7 ng/mL. The detection rates observed in the study are comparable to those reported in targeted biopsy series, despite the absence of mpMRI utilization. It is plausible that incorporation of mpMRI could further enhance diagnostic performance in the setting. Notably, most comparative studies have not reported detailed cost data.

Complication profiles of TRUS-guided and targeted biopsy techniques appear broadly comparable. Although targeted biopsy often involves fewer cores and may theoretically reduce complication risk, minor complications such as hematuria, dysuria, rectal bleeding, and prostatitis remain common in both approaches and are generally managed conservatively. Serious infectious complications, including sepsis, are rare.^19^ Song et al^20^ reported no significant difference in overall complication rates between TRUS-guided biopsy and MRI-TRUS fusion biopsy. Transperineal approaches have been associated with lower infectious risk; however, technical complexity and anesthesia requirements may limit widespread adoption.^21^ El-Achkar et al^22^ observed lower urinary tract infection rates in transperineal biopsy compared with transrectal biopsy (*P* = .006), although rates of hematuria, urinary retention, and sepsis requiring hospitalization were similar between groups. In centers lacking transperineal infrastructure, transrectal biopsy remains a pragmatic and effective option when appropriate patient selection and preparation protocols are applied.

Current guidelines recommend ultrasound-guided biopsy as a standard diagnostic approach while acknowledging the advantages of MRI-targeted biopsy in appropriately equipped centers. Transperineal biopsy has demonstrated improved detection of anterior tumors and reduced infection risk in some series, leading to stronger guideline endorsement.^23^^,^^24^ However, a recent meta-analysis by Wu et al^25^ reported no significant difference in cancer detection rates between transrectal and transperineal ultrasound-guided approaches.

Direct head-to-head cost comparisons between standard and targeted biopsy techniques are limited. In a study conducted in Singapore, Cheng et al^26^ reported a mean cost of US $2223 for MRI-targeted biopsy compared with US $1108 for standard biopsy. Similarly, in a study from the United States, Yun et al^27^ reported mean costs of US $474 for standard biopsy and US $723 for MRI-guided biopsy. Targeted biopsy techniques, particularly MRI-guided approaches, are known to be disadvantaged in terms of imaging requirements, procedural time, and overall economic burden.^28^

When the literature is considered as a whole, standard ultrasound-guided biopsy appears to be comparable to modern targeted biopsy techniques in terms of diagnostic effectiveness and complication profiles. However, TRUS-guided biopsy offers substantial advantages with respect to procedural simplicity, accessibility, cost, and time efficiency.

Several limitations should be acknowledged. The 2 biopsy techniques were not performed within the same institution, and targeted biopsy cost data were derived from private centers due to the absence of national reimbursement codes, potentially introducing variability. Additionally, long-term outcomes of patients with benign biopsy results were not available.

Despite these limitations, to current knowledge, this study represents one of the few analyses comparing TRUS-guided biopsy and targeted biopsy in terms of both diagnostic performance and cost. Moreover, the findings are particularly relevant for countries with limited economic resources, as they demonstrate that TRUS-guided biopsy is substantially more cost-effective than targeted biopsy while achieving comparable diagnostic effectiveness.

## Conclusion

TRUS-guided prostate biopsy remains an effective, accessible, and highly cost-efficient diagnostic modality for patients with suspected PCa. Its ease of implementation, short learning curve, and minimal infrastructure requirements support continued clinical utility. Until barriers related to cost, accessibility, and technical infrastructure for targeted biopsy are adequately addressed, TRUS-guided biopsy is likely to retain an important role in routine clinical practice.

### Artificial Intelligence Usage Statement:

The authors declared that no Artificial Intelligence Tool was used in the preparation of the manuscript.

## Figures and Tables

**Table 1.** Distribution of Comorbid Diseases in Patients

**Comorbidity**	**n**	%
Diabetes mellitus	36	16.2
Pulmonary disease	14	6.3
Hypertension	85	38.4
Dyslipidemia	28	12.6
Psychiatric disease	17	7.6
Neurological disease	12	5.4

**Table 2.** Pathological Outcomes of Biopsy

**Pathology Result**	**n (%)**	**95% CI**
Malignant	89 (40.3)	33.7-47.1
Benign	117 (52.9)	46.1-59.7
ASAP	12 (5.4)	2.8-9.3
Malignancy not excluded	3 (1.4)	0.3-3.9

ASAP, atypical small acinar proliferation.

**Table 3.** Comparison of PSA Values in Benign and Malignant Groups

**Group**	**Median PSA, ng/mL**	**IQR**	*P*
Benign	7.94	4.59	
Malignant	7.84	38.55	.08^a^

PSA, prostate-specific antigen; IQR, interquartile range.

^a^Mann–Whitney *U*-Test.

**Table 4.** Digital Rectal Examination Findings and Comparison in Benign and Malignant Groups

**Group**	**Positive DRE, n (%)**	**Negative DRE, n (%)**	*P*
Benign	36 (30.8)	81 (69.2)	
Malignant	46 (51.7)	43 (48.3)	.002^a^

DRE, digital rectal examination.

^a^χ^2^ test.

**Table 5.** Cost of Prostate Biopsy

**Biopsy Type**	**Mean Cost, US $**
TRUS-guided prostate biopsy	26.8
Targeted biopsy	1449

TRUS, transrectal ultrasound.

## Data Availability

The data that support the findings of this study are available on request from the corresponding author.
